# Supplementary material for: Enhancing protective microglial activities with a dual function TREM2 antibody to the stalk region
Source: EMBO Mol Med. 2020 Mar 10;12(4):e11227. doi: 10.15252/emmm.201911227 (PMC7136959; doi:10.15252/emmm.201911227)
Supplement: Supplementary file 1 — Table EV1 [file EMMM-12-e11227-s001.pdf]

Table EV 1: Antibodies for immunoblotting

For immunoblot detection, the following antibodies were used:

Antigen	Clonality	Species	Clone ID and/or product #	SDS-Gel type	1° antibody dilution	Source	Reference
Mouse TREM2 N-term	Monoclonal	Rat	5F4	12% and 10% Tris-Glycine	varying	Feederle	Xiang et al. EMBO Mol Med 2016
Residues 66-81 of APP	Monoclonal	Mouse	22C11, MAB348	12% Tris-Glycine	1:1000	Merck	-
Calnexin C-term	Polyclonal	Rabbit	SPA-860	12% Tris-Glycine	1:5000	Enzo	-
Residues 1-16 of Aβ	Monoclonal	Rat	2D8	12% and 10% Tris-Glycine	1:100		Shirotani et al. Neurobiol Dis 2007
Human TREM2 N-term	Polyclonal	Goat	AF1828	12% Tris-Glycine	1:2000	R&D Systems	-
Mouse TREM1 N-term	Polyclonal	Goat	AF1187	12% Tris-Glycine	0.5 µg/ml	R&D Systems	-
Mouse TREM2 N-term	Monoclonal	Rat	4D9	12% Tris-Glycine	0.5 µg/ml	Feederle	This publication

Secondary antibodies were HRP-conjugated goat anti-rat IgG (1:10,000; AP136P; Merck), HRP-conjugated goat anti-rabbit IgG (1:10,000; W401B; Promega), HRP-

conjugated goat anti-mouse IgG (1:10,000; W402B; Promega), and HRP-conjugated donkey anti-goat IgG (1:10,000; 705-035-003; Dianova).
