# Supplementary material for: Enhancing protective microglial activities with a dual function TREM2 antibody to the stalk region
Source: EMBO Mol Med. 2020 Mar 10;12(4):e11227. doi: 10.15252/emmm.201911227 (PMC7136959; doi:10.15252/emmm.201911227)
Supplement: Supplementary file 3 — Source Data for Figure 1 [file EMMM-12-e11227-s002.pdf]

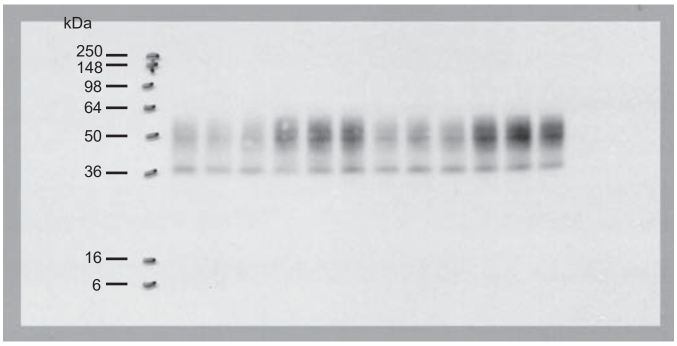

Fig. 1B; TREM2; 12 % Tris Glycine

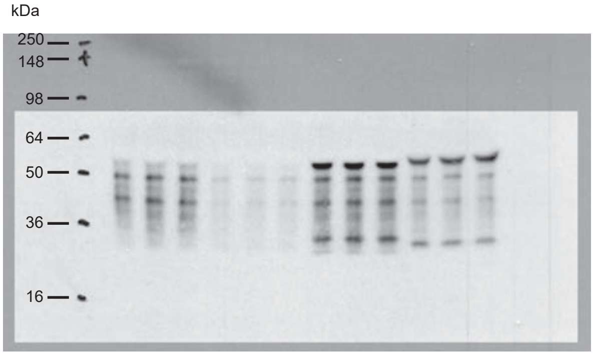

Fig. 1C; TREM2; 10 % Tris Glycine

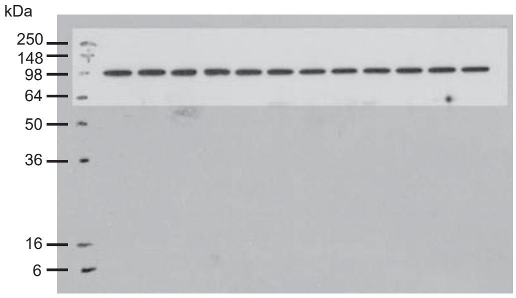

Fig. 1B; calnexin; 12 % Tris Glycine

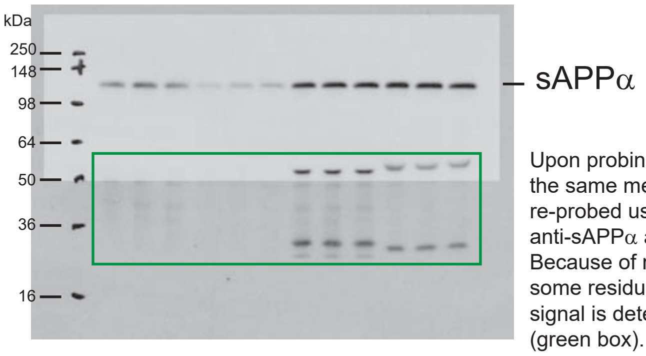

Upon probing for TREM2 the same membrane was re-probed using the anti-sAPPα antibody. Because of re-probing some residual TREM2 signal is detectable (green box).

Fig. 1C; sAPPα; 10 % Tris Glycine

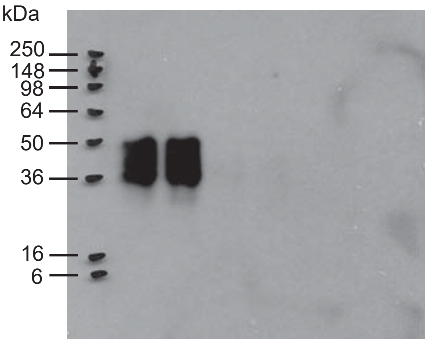

Fig. 1F
TREM2 (human)
12 % Tris Glycine

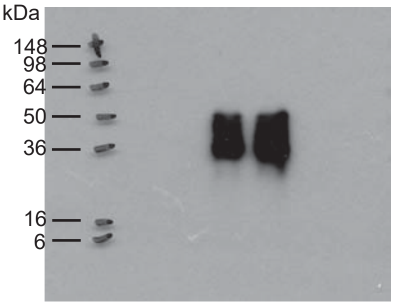

Fig. 1F
TREM2 (mouse)
12 % Tris Glycine

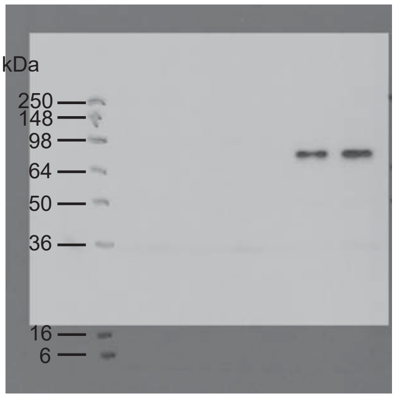

Fig. 1F
TREM1 (mouse)
12 % Tris Glycine

Fig. 1B, 1C, and 1F Source data
