# Supplementary material for: Enhancing protective microglial activities with a dual function TREM2 antibody to the stalk region
Source: EMBO Mol Med. 2020 Mar 10;12(4):e11227. doi: 10.15252/emmm.201911227 (PMC7136959; doi:10.15252/emmm.201911227)
Supplement: Supplementary file 4 — Source Data for Figure 3 [file EMMM-12-e11227-s003.pdf]

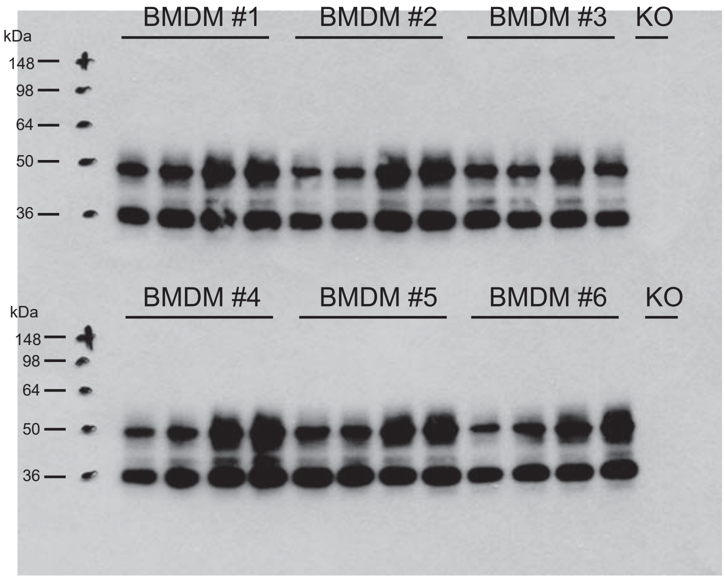

Fig. 3B; TREM2; 12 % Tris Glycine

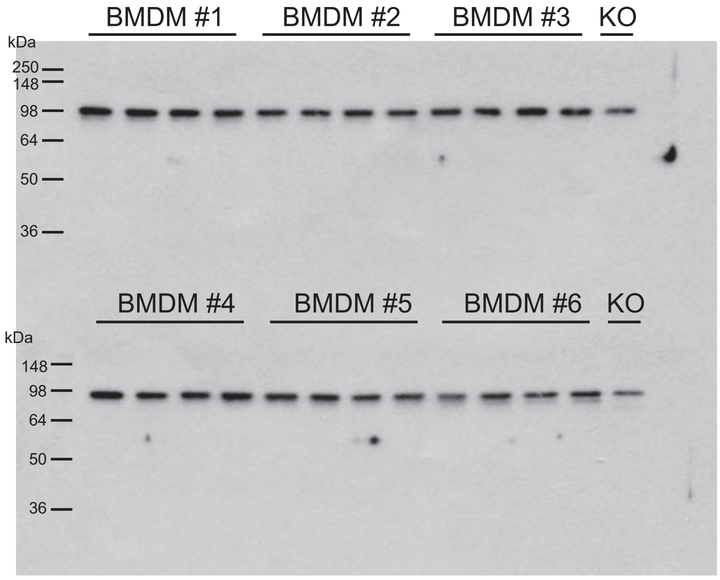

Fig. 3B; calnexin; 12 % Tris Glycine

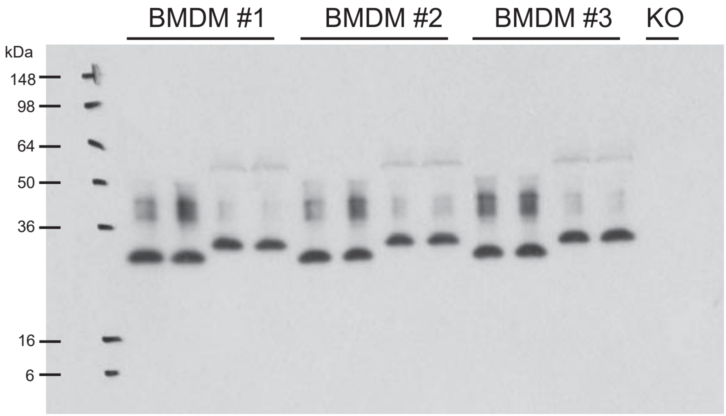

Fig. 3C; TREM2; 12 % Tris Glycine

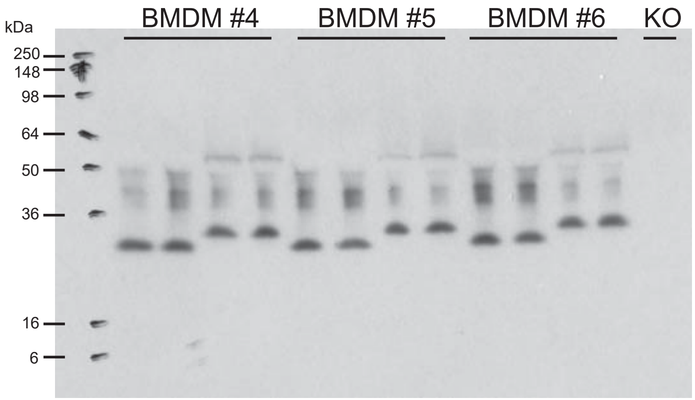

Fig. 3C; TREM2; 12 % Tris Glycine

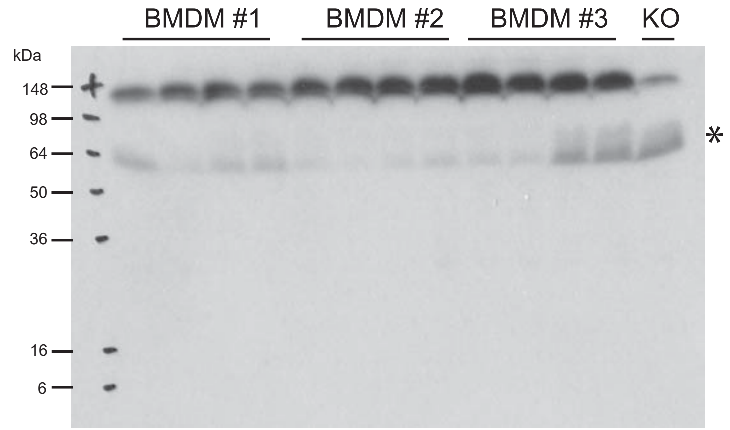

Fig. 3C; APPs; 12 % Tris Glycine

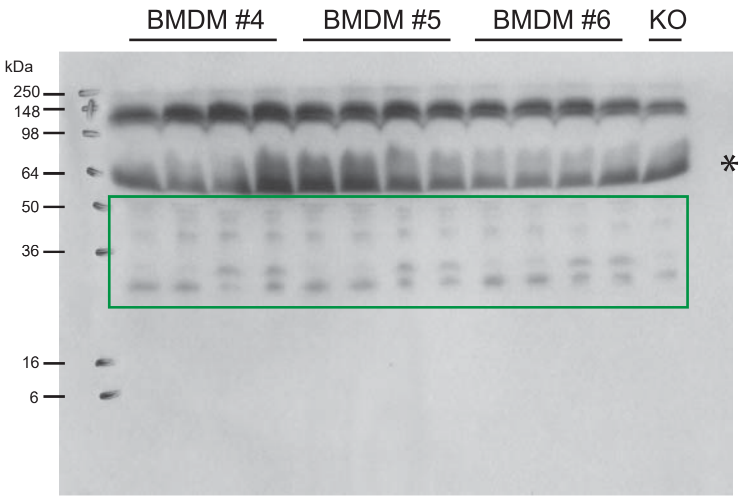

Fig. 3C; APPs; 12 % Tris Glycine

When re-probing for APPs serum components within the cell culture media were detected unspecifically (*). Moreover, in one of the two blots there was some residual TREM2 signal detectable (green box).

Fig. 3B and 3C Source data
