# Supplementary figures and images for: Enhancing protective microglial activities with a dual function TREM2 antibody to the stalk region
Source: EMBO Mol Med. 2020 Mar 10;12(4):e11227. doi: 10.15252/emmm.201911227 (PMC7136959; doi:10.15252/emmm.201911227)

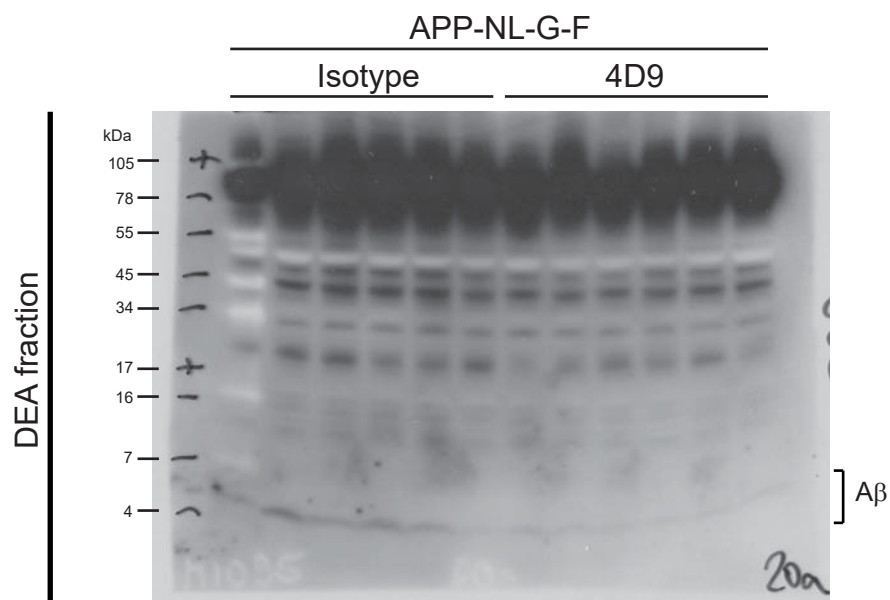

Fig. 7F; A $\beta$ ; 10-20 % Tris Tricine

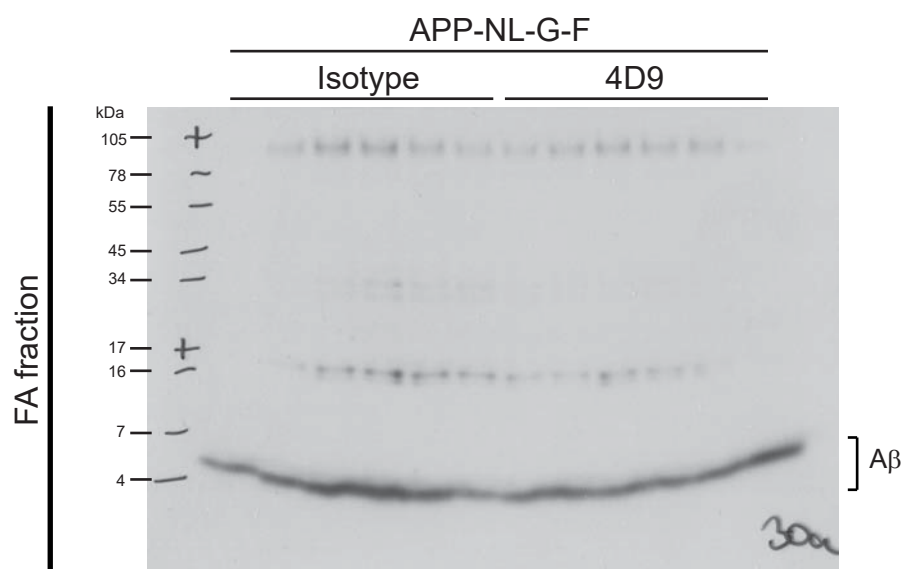

Fig. 7F; A $\beta$ ; 10-20 % Tris Tricine

Supplement: Supplementary file 5 — Source Data for Figure 7 [file EMMM-12-e11227-s004.pdf]
